# Fibroblast growth factor 23 weakens chemotaxis of human blood neutrophils in microfluidic devices

**DOI:** 10.1038/s41598-017-03210-0

**Published:** 2017-06-08

**Authors:** Ke Yang, Hagit Peretz-Soroka, Jiandong Wu, Ling Zhu, Xueling Cui, Michael Zhang, Claudio Rigatto, Yong Liu, Francis Lin

**Affiliations:** 1 Institute of Applied Technology, Hefei Institutes of Physical Science, Chinese Academy of Sciences, Hefei, Anhui P.R. China; 2 Department of Physics and Astronomy, University of Manitoba, Winnipeg, MB Canada; 3 Department of Genetics, Jilin University, Jilin Sheng, China; 4 Seven Oaks General Hospital, Winnipeg, MB Canada; 5 Department of Biosystems Engineering, University of Manitoba, Winnipeg, MB Canada; 6 Department of Immunology, University of Manitoba, Winnipeg, MB Canada; 7 Department of Biological Sciences, University of Manitoba, Winnipeg, MB Canada

## Abstract

Neutrophil trafficking in tissues critically regulates the body’s immune response. Neutrophil migration can either play a protective role in host defense or cause health problems. Fibroblast growth factor 23 (FGF23) is a known biomarker for chronic kidney disease (CKD) and was recently shown to impair neutrophil arrest on endothelium and transendothelial migration. In the present study, we further examined the effect of FGF23 on human blood neutrophil chemotaxis using two new microfluidic devices. Our results showed that chemotaxis of FGF23 pre-treated neutrophils to a *f*MLP gradient, in the presence or absence of a uniform FGF23 background, is quantitatively lower compared to the control cells. This effect is accompanied with a stronger drifting of FGF23 pre-treated cells along the flow. However, without the FGF23 pre-treatment, the FGF23 background only reduces chemotaxis of transmigrated cells through the thin barrier channel to the *f*MLP gradient. The effect of FGF23 on neutrophil migration and the correlation between multiple cell migration parameters are further revealed by chemotactic entropy and principle component analysis. Collectively, these results revealed the effect of FGF23 on weakening neutrophil chemotaxis, which shed light on FGF23 mediated neutrophil migration with direct disease relevance such as CKD.

## Introduction

Cell migration significantly contributes to physiological and pathological processes such as host defense^1, 2^, embryonic development^3^, wound healing^4^ and cancer metastasis^5^. Neutrophils are the most abundant leukocytes and are considered primarily important for the body’s innate immune system^6^. Incorrectly signaled neutrophil chemotaxis can lead to various cellular malfunctions such as autoimmune diseases and fatal disorders^2^. Acute or chronic autoimmune diseases such as asthma and chronic obstructive pulmonary disease (COPD) are long known to result from elevated neutrophil migration and infiltration to the airway^7, 8^. By contrast, neutrophil migration and trafficking are impaired in other diseases such as sepsis and chronic kidney disease (CKD)^[9–11^. Recently, tumor-associated neutrophils were identified, which can play dual (anti-tumor or pro-tumor) roles to mediate cancer development^12^.

A recent work showed that fibroblast growth factor 23 (FGF23) can inhibit various neutrophil trafficking related functions such as adhesion, integrin activation and transendothelial migration^11^. The impaired neutrophil recruitment and host defense by FGF23 were further demonstrated *in-vivo* in CKD models^11^. CKD is a common chronic disease with high incidence in both developed and developing regions. The serum FGF23 level in CKD patients is significantly elevated^13^. CKD patients have an increased risk of bacterial infections due to immune suppression^14–16^, which further complicates kidney transplantation therapy^17^. FGF23 mediates various diseases through the FGF receptor (FGFRs) signaling in the target cells^11, 18–21^. Particularly, FGF23 was suggested to be a regulator for innate immunity^22^ and human neutrophils express varying levels of FGFRs in their cytosol and on their cytoplasmic membrane to interact with FGF23^23^. Collectively, these previous findings argue the importance to further investigate FGF23 mediated neutrophil function and its relevance to health problems. In particular, we further hypothesize that FGF23 can affect neutrophil chemotaxis, which was investigated in this study.

**Figure 1:**
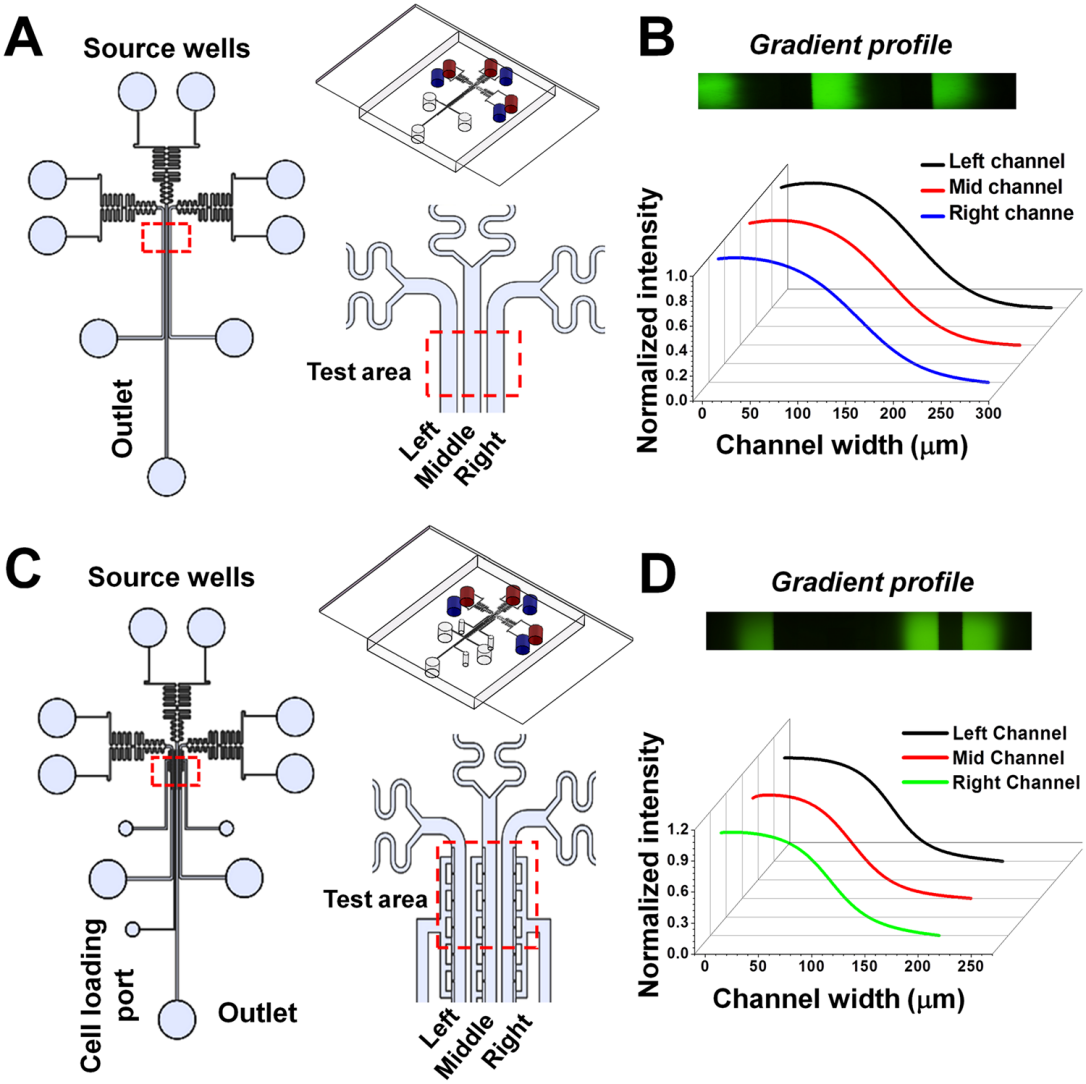
Illustration of the C^3^-Chip and D^3^-Chip. (**A**) Illustration of the C^3^-Chip design. The enlarged view shows the test area of the C^3^-Chip. The 3D view of the device is also shown. For a single chip, three gradient generating units are configured in parallel. Each unit has its independently controlled source wells and outlet. Cells can be loaded to each unit through the unit specific outlet. The test area (labeled by the red box) is the imaging field of the three parallel gradient channels (left, middle and right); (**B**) Identical gradient generation in the three parallel units of the C^3^-Chip; (**C**) Illustration of the D^3^-Chip design. The enlarged view shows the test area of the D^3^-Chip. The 3D view of the device is also shown. Similar to the C^3^-Chip, for a single D^3^-Chip, three gradient generating units are configured in parallel. Each unit has its independently controlled source wells, outlet and cell loading port. The test area (labeled by the red box) is the imaging field of the three parallel gradient channels (left, middle and right). The cell docking principle was described in details previously^28^ and illustrated in Fig. S1B; (**D**) Identical gradient generation in the three parallel units of the D^3^-Chip. In the gradient plots, the edge of the gradient channel by the barrier channel for each unit is defined as the reference point. Thus, the gradient direction in the right unit is opposite to it in the left and middle units in the D^3^-Chip.

Over the last two decades, microfluidic devices have been increasingly used for studying cell migration and chemotaxis owing to their advantages in miniaturization and micro-environmental control^24, 25^. More recently, the microfluidics approach has been applied to developing diagnostic applications for neutrophil migration related diseases^26, 27^. We have previously developed a standalone microfluidic gradient generator to study neutrophil chemotaxis induced by COPD patients’ sputum samples^8^. Furthermore, we developed an all-on-chip method for rapid neutrophil chemotaxis test by combining a microfluidic gradient generating device with the cell docking feature and an on-chip magnetic cell separation module directly from whole blood^28^. These previous developments built the background microfluidic technology for advanced cell migration and chemotaxis studies. On the other hand, these devices can only run a single cell migration experiment at a time, which limits the throughput for parallel comparison of different conditions. For this reason, we in this study further developed a higher throughput version of each device, i.e. a triple channel device (C^3^-Chip) and a triple docking device (D^3^-Chip). The C^3^-Chip was used for parallel comparison of human blood neutrophil chemotaxis under three different experimental conditions on a single device (Fig. 1A). The D^3^-Chip further incorporated a thin barrier channel design for each of the three test units on the same device to partially mimic transmigration of human blood neutrophils and to pre-align cells in the device before the gradient exposure (Figs 1C and S1B). In addition to the commonly used chemotaxis parameters such as chemotactic index and cell speed, we further applied the entropy analysis and the data mining approach to quantify the altered neutrophil migratory behaviors resulting from FGF23 exposure.

## Results

### Development of the C^3^-Chip and the D^3^-Chip

The basic designs of our triple-unit microfluidic devices were based on the pervious single-unit devices^8, 28^. The C^3^-Chip and the D^3^-Chip were further designed to improve experimental throughput, which is critical for addressing the specific biological questions. Dimensions of the design were optimized so all three test units fit into a single field of view (FOV) in our microscope. Such a design allows time-lapse imaging of all three test units without moving the stage. These devices can be further developed to allow higher throughput by more compact designs and the use of a programmable motorized microscope stage for more advanced experiments. Moreover, both the C^3^-Chip and the D^3^-Chip devices allow rapid independently-controlled pump-less stable chemical gradient generation (Figs 1 and S1). The gradient profile is stable over time in each channel for up to 1 hr and allows identical gradient generation in all three channels (Figs 1 and S1). Here we measured the gradients at 1.5 mm below the junction of the inlet channels. Each unit has its specific inlets, outlets and cell loading port and thus permits parallel cell migration experiments under different conditions. For the C^3^-Chip, cells are randomly seeded in the gradient channel (Fig. S1A). The D^3^-Chip incorporated the additional cell docking structure based on multi-height channels to align the cells next to the thin barrier channel (Fig. S1B). Cells were initially trapped in the docking area as their size before gradient stimulation is larger than the barrier channel height. Cells need to deform and squeeze through the thin barrier channel (3 μm high and 20 μm wide) to migrate into the gradient channel upon gradient application. This cell alignment method unifies the cells’ initial positions relative to the gradient, which improves the accuracy of cell migration and chemotaxis measurement. Furthermore, cell migration through the barrier channel in response to a chemoattractant gradient partially mimics transmigration of cells through blood vessel barrier into tissues. For both C^3^-Chip and D^3^-Chip, cell loading to all three test units and gradient generation are quick and consistent. The time gap between the setup of different test units is minimal. These features are important to allow more reliable cell migration test results.

### FGF23 affects human neutrophil chemotaxis in the C^3^-Chip

**Figure 2:**
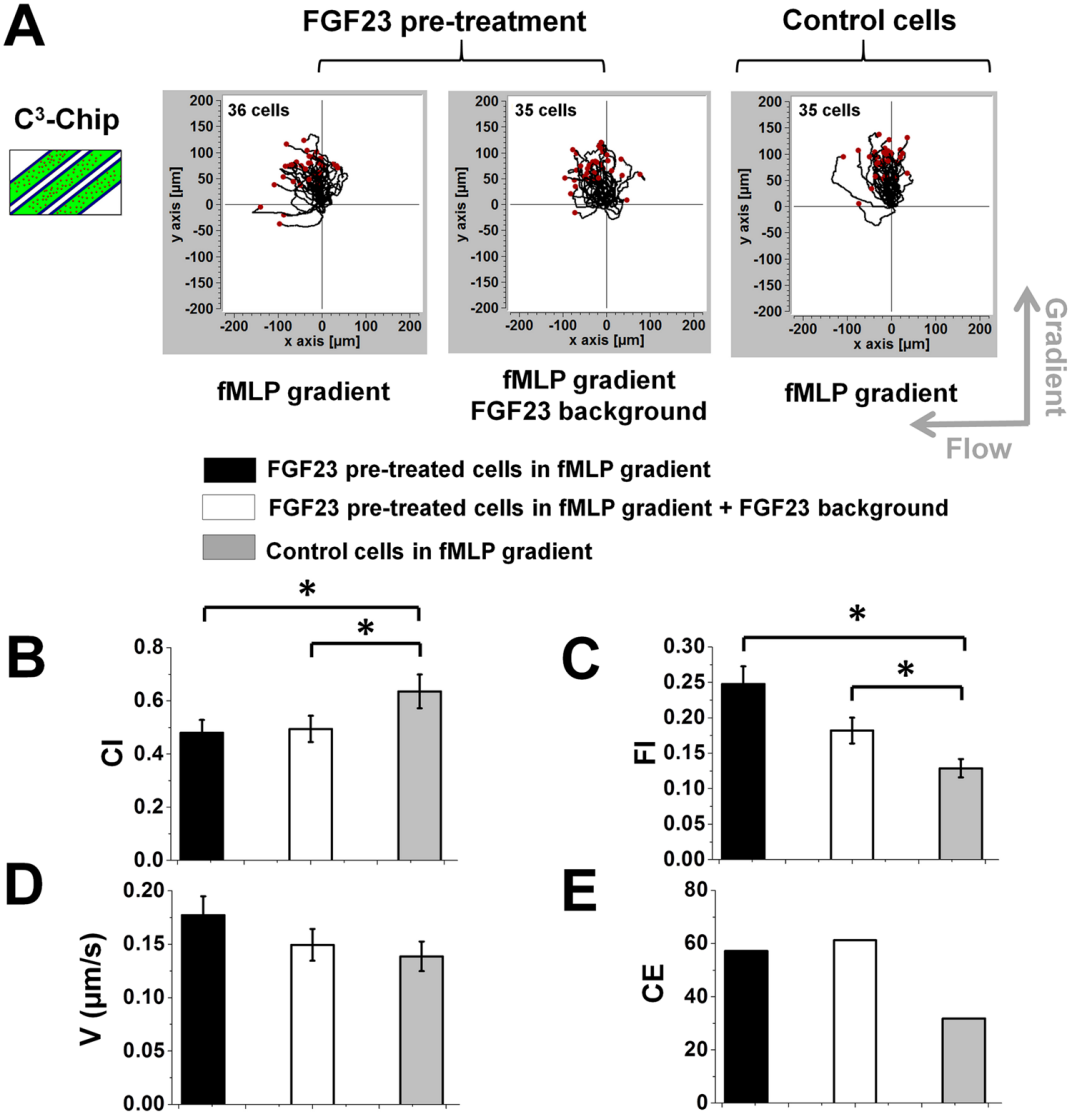
Chemotaxis of FGF23 pre-treated cells and un-treated control cells in the C^3^-Chip. (**A**) Cell migration tracks of FGF23 pre-treated cells in a 100 nM *f*MLP gradient with or without a FGF23 uniform background (left two plots) and cell migration tracks of un-treated control cells in a 100 nM *f*MLP gradient (right plot) in the C^3^-Chip. The cell tracks are normalized to have the same starting point in the plot. The gradient direction, the flow direction and the number of cells for each plot are indicated; (**B**–**E**) Comparison of quantitative cell migration parameters including chemotactic index (CI), flowtactic index (FI), speed (V) and chemotactic entropy (CE). The data for each parameter are presented as the average of all cells from representative experiments. The error bar indicates the standard error of the mean. *Indicates *p* < 0.05 for comparison between specific conditions using the Student’s *t*-test.

**Figure 3:**
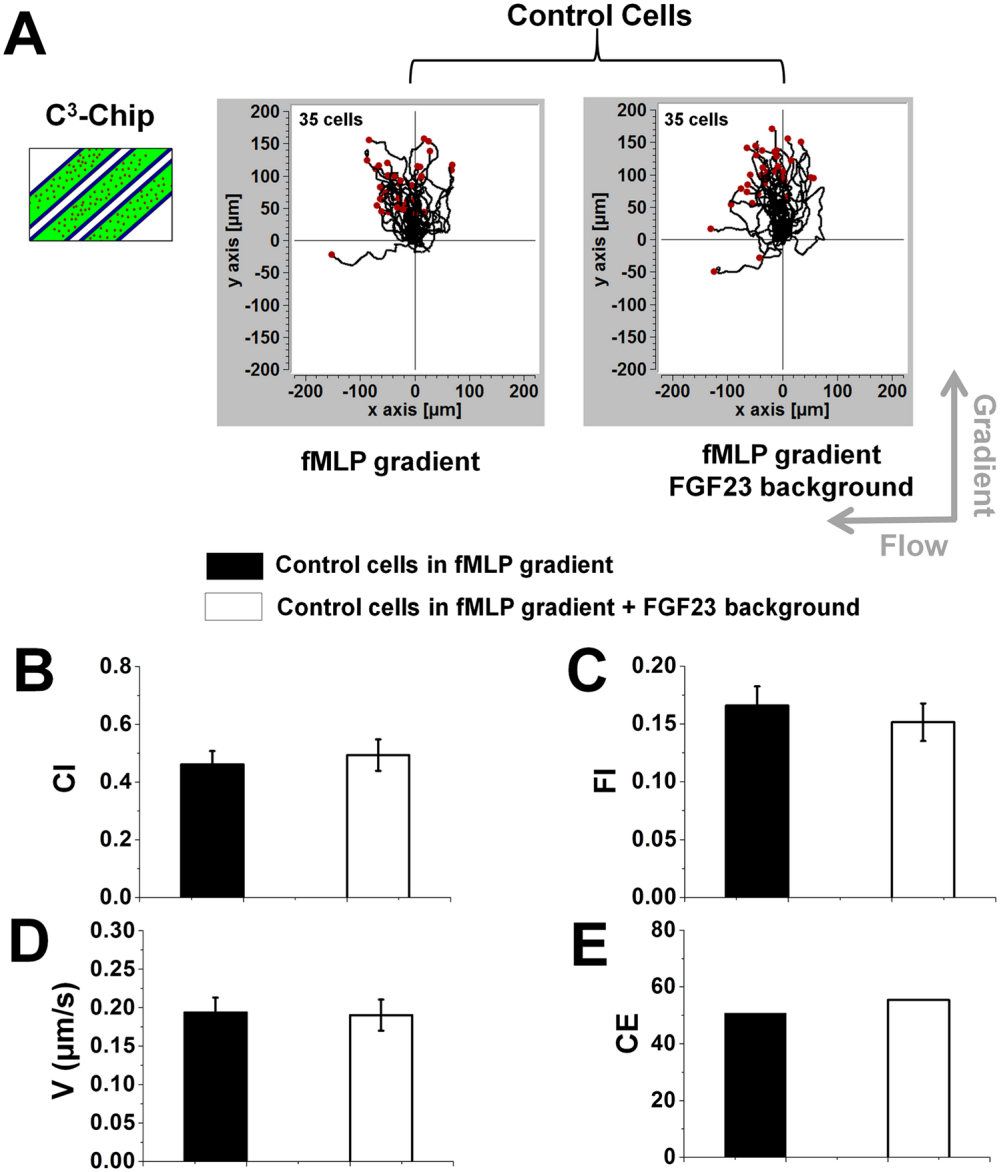
Chemotaxis of un-treated control cells with or without active FGF23 exposure in the C^3^-Chip. Cell migration tracks of control cells in a 100 nM *f*MLP gradient with (right plot) or without (left plot) a FGF23 uniform background in the C^3^-Chip. The cell tracks are normalized to have the same starting point in the plot. The gradient direction, the flow direction and the number of cells for each plot are indicated; (**B**–**E**) Comparison of quantitative cell migration parameters including chemotactic index (CI), flowtactic index (FI), speed (V) and chemotactic entropy (CE). The data for each parameter are presented as the average of all cells from representative experiments. The error bar indicates the standard error of the mean.

Using the C^3^-Chip, we evaluated the effect of FGF23 on human neutrophil chemotaxis to a *f*MLP gradient. Cell migration was tested under three different conditions including: (1) FGF23 pre-treated cells in the *f*MLP gradient; (2) FGF23 pre-treated cells in the *f*MLP gradient with a uniform FGF23 background; (3) un-treated control cells in the *f*MLP gradient (Fig. 2). Our results showed that under all conditions, neutrophils effectively migrated toward the *f*MLP gradient (Fig. 2). Quantitative cell migration analysis showed that the FGF23 pre-treatment resulted in a significant decrease of neutrophil chemotaxis (as measured by CI) and potentiated flowtaxis (as measured by FI). Using a similar experimental design, chemotaxis of un-treated control cells to the *f*MLP gradient in the presence or absence of a FGF23 background was compared (Fig. 3). In contrast to the FGF23 pre-treatment experiments, our results showed comparable chemotaxis and flowtaxis (as measured by CI and FI respectively) of un-treated cells to the *f*MLP gradient with or without a uniform FGF23 background. In both sets of experiments, cell speeds are comparable among different conditions (Figs 2 and 3). Collectively, the results from the C^3^-Chip experiments showed that FGF23 pre-treatment can passively decrease neutrophil chemotaxis to *f*MLP. The uniform FGF23 background in the *f*MLP gradient without the FGF23 pre-treatment is not sufficient to influence neutrophil chemotaxis in the C^3^-Chip experiments. The accompanied flowtaxis effect and the un-affected cell migration speed suggest that the weakened chemotaxis results from at least the altered chemotactic migration direction of FGF23 pre-treated cells. The decrease of chemotaxis is coupled to the increase of flowtaxis. When cell migration is more affected by the flow, chemotaxis will be less effective for cells to reach the target. Consistently, entropy analysis showed the increased disorder of chemotactic migration direction in the FGF23 pre-treated cells comparing to the control cells (Fig. 2E). No significant difference of CE was found without FGF23 pre-treatment (Fig. 3E). The baseline level of CI (close to 0), FI (close to 0), and speed (<0.1 μm/s) in the medium control is significantly lower than the chemotaxis group. CE (>100) is significantly higher in the medium control than the chemotaxis group.

It is worth pointing out the variation of cell migration parameters under the control condition in the two sets of experiments (Figs 2 and 3) due to the following reasons. First, primary neutrophils are expected to have variations in their chemotaxis ability from different healthy blood donors. Second, a main limitation of the C^3^-Chip is that cells are seeded randomly in the gradient channel. Therefore, there is no control of their starting positions in the gradient. Thus, the exact values of these cell migration parameters in different devices are not expected to be the same. The effect of FGF23 treatment on cell migration is measured relative to the internal control condition (i.e. un-treated control cells in a *f*MLP gradient) on the same device, which is enabled by the high-throughput format of this C^3^-Chip. The limitation of the C^3^-Chip in cell loading motivated us to further test the effect of FGF23 on neutrophil migration using the D^3^-Chip.

### FGF23 affects human neutrophil transmigration and chemotaxis in the D^3^-Chip

**Figure 4:**
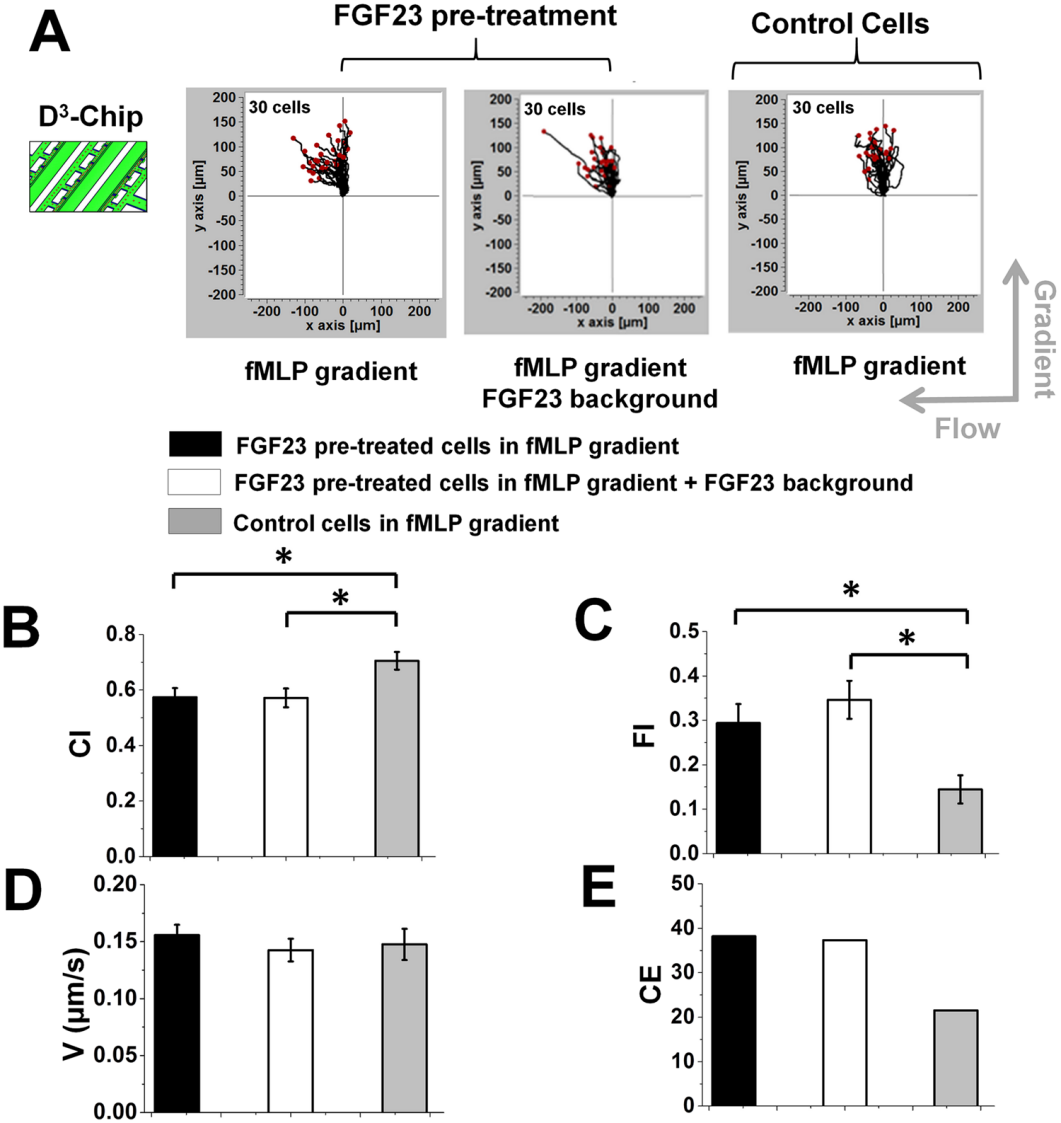
Chemotaxis of FGF23 pre-treated cells and un-treated control cells in the D^3^-Chip. (**A**) Cell migration tracks of FGF23 pre-treated cells in a 100 nM *f*MLP gradient with or without a FGF23 uniform background (left two plots) and cell migration tracks of control cells in a 100 nM *f*MLP gradient (right plot) in the D^3^-Chip. The cell tracks are normalized to have the same starting point in the plot. The gradient direction, the flow direction and the number of cells for each plot are indicated; (**B**–**E**) Comparison of quantitative cell migration parameters including chemotactic index (CI), flowtactic index (FI), speed (V) and chemotactic entropy (CE). The data for each parameter are presented as the average of all cells from representative experiments. The error bar indicates the standard error of the mean. *Indicates *p* < 0.05 for comparison between specific conditions using the Student’s *t*-test.

**Figure 5:**
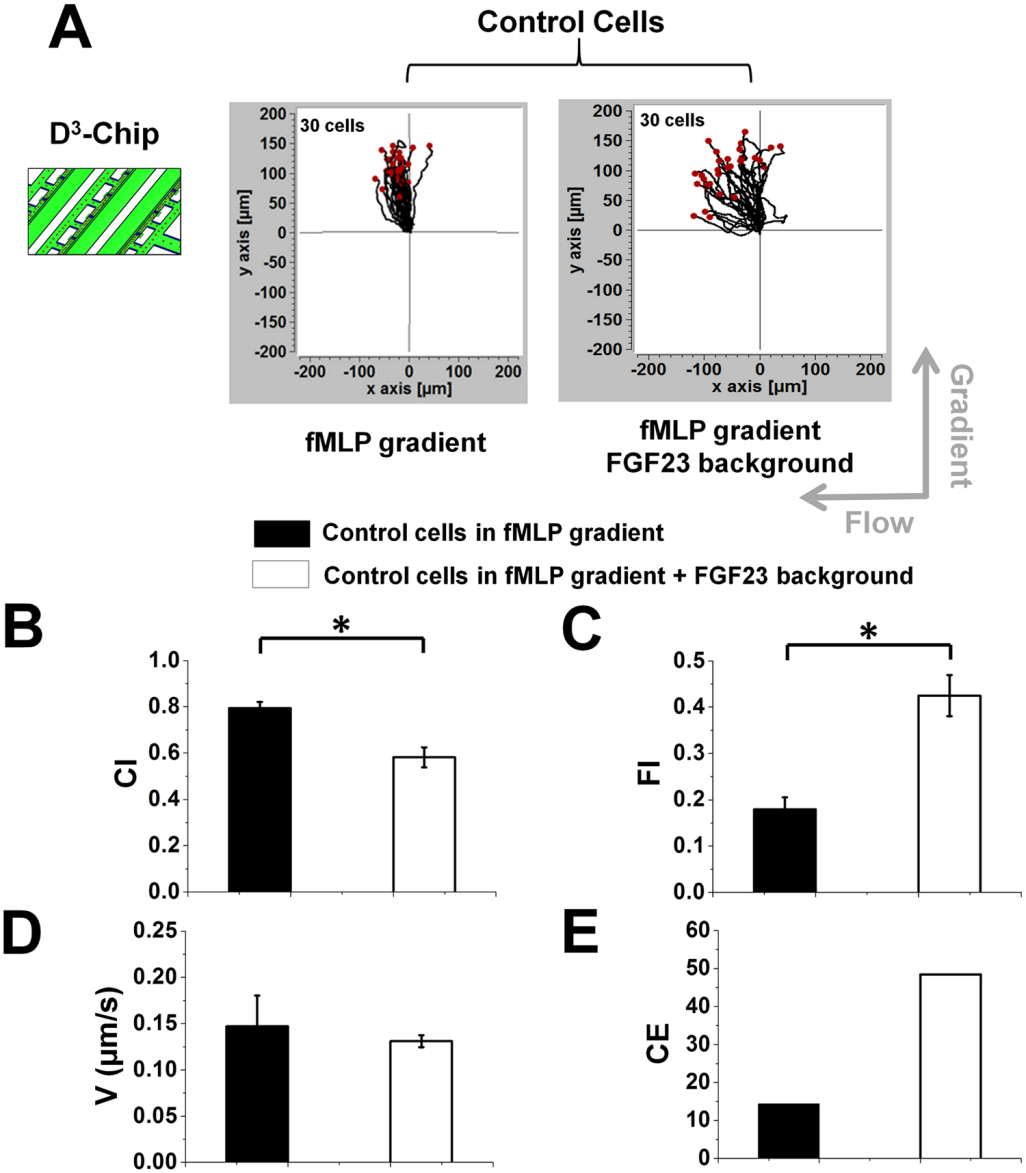
Chemotaxis of un-treated control cells with or without active FGF23 exposure in the D^3^-Chip. Cell migration tracks of control cells in a 100 nM *f*MLP gradient with (right plot) or without (left plot) a FGF23 uniform background in the D^3^-Chip. The cell tracks are normalized to have the same starting point in the plot. The gradient direction, the flow direction and the number of cells for each plot are indicated; (**B**–**E**) Comparison of quantitative cell migration parameters including chemotactic index (CI), flowtactic index (FI), speed (V) and chemotactic entropy (CE). The data for each parameter are presented as the average of all cells from representative experiments. The error bar indicates the standard error of the mean. *Indicates *p* < 0.05 for comparison between specific conditions using the Student’s *t*-test.

Using the D^3^-Chip, we tested the effect of FGF23 on the partially mimicked neutrophil transmigration (without patterning an actual layer of endothelial cells). Experimental configurations mirrored the design in the C^3^-Chip. Our results showed that, under all conditions, neutrophils can effectively transmigrate through the physical barrier of the docking channel into the gradient channel. Consistent with the results in the C^3^-Chip, FGF23 pre-treatment led to quantitatively weakened neutrophil chemotaxis (as measured by CI) and potentiated flowtaxis (as measured by FI)(Fig. 4 **)**. Interestingly, in contrast to the C^3^-Chip, active FGF23 exposure for the un-treated control cells during cell migration experiment (by adding an uniform FGF23 background in the *f*MLP gradient) in the D^3^-Chip also decreased chemotaxis and potentiated flowtaxis (as measured by CI and FI respectively)(Fig. 5). Again, under all conditions, cell speed was comparable (Figs 4 and 5). CE analysis showed the consistent results of increased disorder in chemotactic migration direction in FGF23 treated cells comparing to the control cells (Figs 4E and 5E). Compared with the C^3^-Chip experiments, the results from the D^3^-Chip experiments further showed that passive and active FGF23 treatment can operate independently or in combination to weaken chemotaxis of transmigrated neutrophils to the *f*MLP gradient. The possible reasons concerning the difference of the results between the C^3^-Chip and the D^3^-Chip are discussed later in the Discussion section. It is also worth noting that different cell migration parameters in the control between experiments are more consistent in the D^3^-Chip compared with the C^3^-Chip, which we believe is because of the cell loading control in the D^3^-Chip. On the other hand, the average values of each cell migration parameter still vary in our repeating experiments with the following range: 1) *f*MLP gradient control without FGF23 pre-treatment: the average CI ranges from 0.67 to 0.79; the average FI ranges from 0.14 to 0.26; the average speed ranges from 0.14 μm/sec to 0.18 μm/sec; CE ranges from 10 to 38. 2) *f*MLP gradient with FGF23 pre-treatment: the average CI ranges from 0.48 to 0.57; the average FI ranges from 0.29 to 0.38; the average speed ranges from 0.14 μm/sec to 0.17 μm/sec; CE ranges from 37 to 52. 3) *f*MLP gradient with FGF23 background and FGF23 pre-treatment: the average CI ranges from 0.48 to 0.57; the average FI ranges from 0.34 to 0.45; the average speed ranges from 0.15 μm/sec to 0.17 μm/sec; CE ranges from 38 to 49. 4) *f*MLP gradient with FGF23 background but without FGF23 pre-treatment: the average CI ranges from 0.49 to 0.63; the average FI ranges from 0.31 to 0.42; the average speed ranges from 0.14 μm/sec to 0.18 μm/sec; CE ranges from 37 to 59.

For both the C^3^-Chip and the D^3^-Chip experiments, we tested different FGF23 pre-treatment time ranging from 1 hr to 4 hrs and found similar effect in chemotaxis experiment. Thus, the data presented in this paper are from 1 hr FGF23 pre-treatment for consistency.

### Principle component analysis confirms the effect of FGF23 on neutrophil chemotaxis

**Figure 6:**
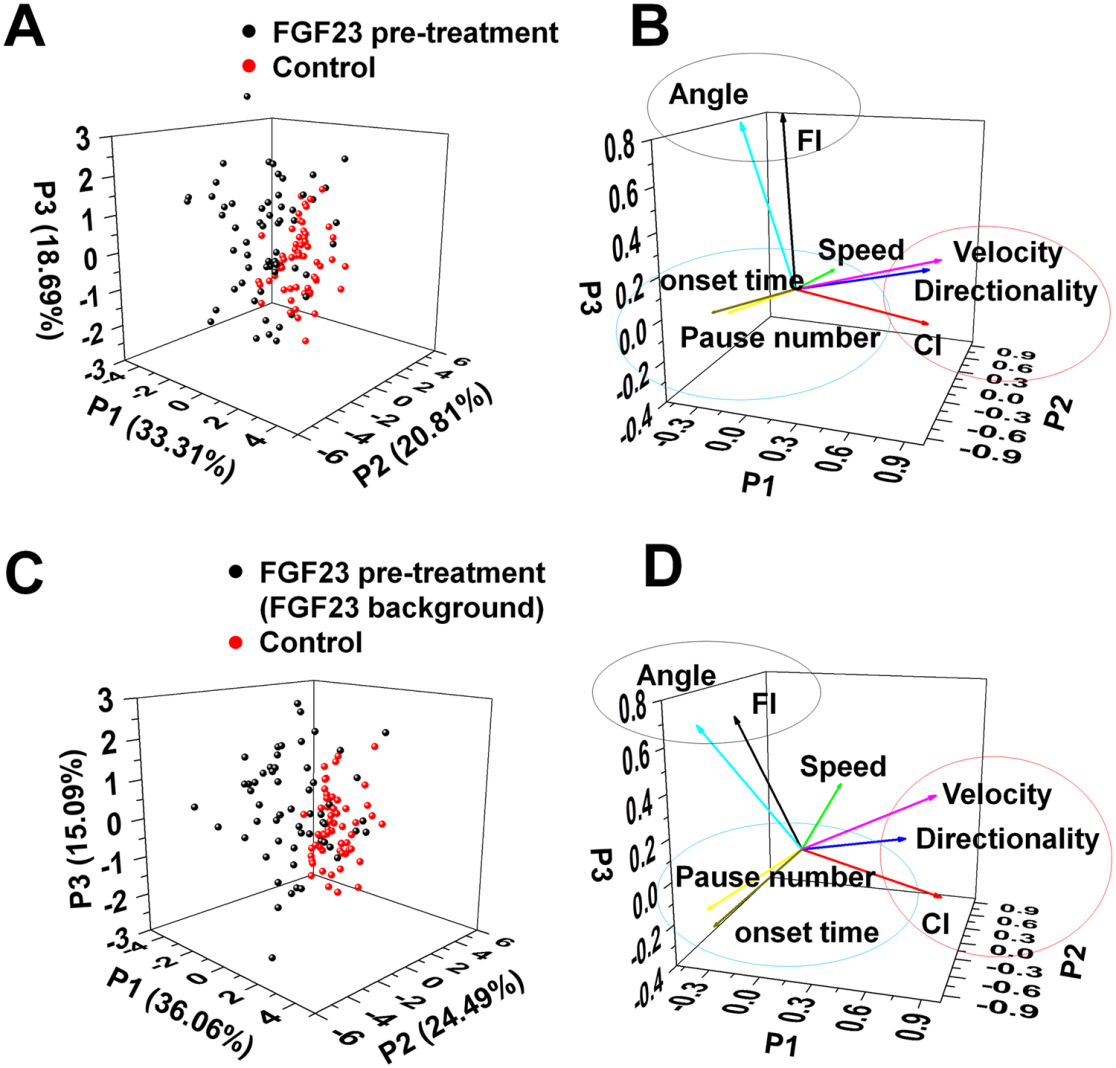
Principle component analysis to compare cell migration with or without FGF23 treatment. (**A**) Score plot of FGF23 pre-treated cells (black dots) and un-treated control cells (red dots) in a 100 nM *f*MLP gradient in the 3D transformed domain of the first three principle component axes (P1, P2, P3). The percentage of variation accounted by each principle axis is labeled; (**B**) Correlation vector plot of cell migration parameters in the 3D transformed domain of the first three principle component axes (P1, P2, P3) for the experiments in (**A**). The vector indicates the correlation of each cell migration parameter to the first three principle component axes. Significantly correlated parameters are circled; (**C**) Score plot of FGF23 pre-treated cells and control cells in a *f*MLP gradient with a uniform FGF23 background in the 3D transformed domain of the first three principle component axes (P1, P2, P3). The percentage of variation accounted by each principle axis is labeled; (**D**) Correlation vector plot of cell migration parameters in the 3D transformed domain of the first three principle component axes (P1, P2, P3) for the experiments in (**C**). The vector indicates the correlation of each cell migration parameter to the first three principle component axes. Significantly correlated parameters are circled. The data shown are from representative experiments using the C^3^-Chip.

Single cell migration parameter analysis showed that FGF23 weakens but does not inhibit human blood neutrophil chemotaxis. More specifically, CI was relatively high in both the FGF23 treated group and the un-treated control group. Furthermore, flowtaxis (as measured by FI) and the accuracy of chemotactic migration direction (as measured by CE) also demonstrated the altered neutrophil migration by FGF23. These findings suggested that a multi-parameter data mining approach may confirm the effect of FGF23 on neutrophil migration and reveal the relative importance and correlations of different cell migration parameters. Here, we demonstrated the use of PCA, a commonly used unsupervised machine learning method, for analyzing our cell migration data. A panel of cell migration parameters extracted from the cell tracking data including CI, FI, speed, directionality, angle of migration direction, velocity, pause number, and onset time, were used for the PCA (Table 1). Our results showed relatively clear clustering of FGF23 treated cells (black dots) and un-treated control cells (red dots) in the *f*MLP gradients with (Fig. 6C) or without (Fig. 6A) a FGF23 background in the 3D transformed domain by the first three principle component axes. Similar results were found in the 2D plot in the transformed domain of the first two principle component axes (Fig. S2). The relative importance of each cell migration parameter was also reflected by their correlation to the principle component axes (Tables S1 and S2). Furthermore, correlation vector plot of cell migration parameters in the same 3D principle component axis domain revealed consistent correlation between different parameters (Fig. 6B,D). Some considerably correlated parameters include the following: 1) CI, velocity, and directionality are positively correlated; 2) FI and migration angle are positively correlated while CI and migration angle are negatively correlated, which implies the negative correlation between CI and FI; 3) pause number and speed are negatively correlated (Tables S1 and S2). These correlations from PCA are consistent with the experimental observations. Interestingly, onset time and pause number also showed considerable positive correlation in the PCA, especially for the FGF23 pre-treated cells and un-treated control cells in the *f*MLP gradient (Fig. 6A and Table S1). Collectively, the PCA analysis, which integrated multiple cell migration parameters, confirmed the distinct chemotactic migratory properties of neutrophils to *f*MLP with or without FGF23 exposure.

**Table 1:** Cell migration parameters used for PCA.

Parameter	Description
FI	The ratio of the cell displacement in the flow direction to the total migration distance.
CI	The ratio of the cell displacement in the gradient direction to the total migration distance.
Speed	The total migration distance over the experiment period.
Directionality	The ratio of the cell displacement to the total migration distance.
Angle	The angle of the total cell displacement vector with respect to the positive horizontal direction.
Velocity	The ratio of the total cell displacement to the experiment period.
Pause number	The number of times that a cell stopped moving for longer than 30 s during the experiment period.
Onset time	The time period after the gradient is applied and before a cell initiates significant migration (>10 μm).

The names of the parameters are listed in the left column and the corresponding definitions are given in the right column.

## Discussion

In this study, our results showed a general weakening effect of FGF23 on human neutrophil chemotaxis *in vitro*. Chemotaxis of FGF23 pre-treated neutrophils to *f*MLP was weakened in the presence or absence of a FGF23 background when compared to the un-treated control cells. Consistently, the weakened chemotaxis was coupled with potentiated flowtaxis, suggesting the possible altered adhesion of migrating cells to the substrate. These results are in coherent agreement with FGF23 induced impairment of neutrophil adhesion and transendothelial migration upon chemokine activation^11^.

FGF23 pre-treatment alone or in combination with active FGF23 exposure weakened neutrophil chemotaxis in both C^3^-Chip and D^3^-Chip. These results suggest a synergistic effect of passive and active FGF23 treatment on neutrophil chemotactic signaling and migration. Interestingly, active FGF23 treatment of control cells decreased neutrophil chemotaxis to *f*MLP in the D^3^-Chip but not in the C^3^-Chip. The main difference between the two types of devices is the cell docking function in the D^3^-Chip. The cell docking feature mechanically confines the cells to the docking area without requiring firm cell adhesion to the substrate. Therefore, we speculate that in the D^3^-Chip, the *f*MLP gradient can attract cells with relatively high heterogeneous adhesion properties into the gradient channel, and the active FGF23 treatment itself can result in significant weakening effect of neutrophil chemotaxis. In the C^3^-Chip, on the other hand, strong adhesion of cells to the substrate under flow is a prerequisite for cell settlement in the device. Therefore, the active FGF23 treatment itself may not be sufficient to effectively weaken chemotaxis of the attached cells over the short period of the migration assay without FGF23 pre-treatment.

Physiological FGF23 level in blood is associated with different diseases^29–32^. In this study, we aimed to associate the effect of FGF23 in cell migration with CKD ^11^. CKD patients are more susceptible to infections due to immune-suppression^33^ and have a higher risk of cardiovascular related diseases such as diabetes^29, 34^, atherosclerosis^35^ and left ventricular hypertrophy^36^. The FGF23 level in blood of patients with advanced stage CKD is highly elevated, suggesting FGF23 as an important CKD biomarker^37, 38^. Biological links between FGF23 and neutrophils have been consistently identified in CKD such as impaired neutrophil adhesion and transendothelial migration^11^. Exposure of neutrophils to FGF23 can operate through the FGFR-dependent pathways leading to impaired host defense functions. The weakening effect of neutrophil chemotaxis by FGF23 as revealed in this study provides new mechanistic insight of FGF23 mediated neutrophil trafficking in CKD. Further confirmation using CKD patients’ cell samples is required to establish the basis for clinical diagnostic and therapeutic applications.

The effect of FGF23 on neutrophil migration in this study presents a scenario that FGF23 treated cells and control cells have a quantitative difference in chemotaxis. The conventional individual cell migration parameter analysis including chemotactic index and flowtactic index revealed such quantitative difference in a consistent and statistically significant manner. CE analysis further showed that the weakened chemotaxis by FGF23 treatment is associated with less organized chemotactic migratory direction, and therefore higher entropic disorders. The entropy analysis has been previously applied to analyzing collective cell migration based on particle image velocimetry (PIV) data^39, 40^. Here we demonstrate that this technique is also useful for single cell migration tracking data. In addition, the data mining approach has been successfully applied to analyzing biological and biomedical data^41–43^. Our results based on the PCA analysis demonstrated that this learning approach is effective in distinguishing the FGF23 pre-treated cells and un-treated control cells. The PCA can further determine correlations between individual cell migration parameters and their relative importance to interpret the observed different cell migratory behaviors. More cell migration parameters can be added to further enhance the PCA.

In conclusion, we developed two high-throughput microfluidic devices and successfully applied these devices to studying the effect of FGF23 on neutrophil migration and chemotaxis. This study for the first time characterized how FGF23 affects neutrophil chemotaxis, and suggests the potential of targeting FGF23 mediated neutrophil chemotaxis for relevant disease diagnosis and therapy.

## Materials and Methods

All experimental methods and protocols were carried out in accordance with relevant guidelines and regulations set at the University of Manitoba. The biological experiments were carried out under the approved biosafety permit at the University of Manitoba and the ethics approval for obtaining blood samples from healthy human donors (Protocol number: J2015:022) was granted by the Joint-Faculty Research Ethics Board at the University of Manitoba.

### Microfluidic device design and fabrication

In this study, two types of microfluidic devices were fabricated using the standard photolithography and soft-lithography technique^44^. The photomasks of the devices were designed using Solidworks (ver. 2013, Dassault Systemes S.A.) and the design was printed onto a transparency film at 24,000 dpi resolution (Fineline Imaging). The master mold of the C^3^-Chip was fabricated by single-layer photolithography (Fig. 1) and the master mold of the D^3^-Chip with integrated cell docking structure was fabricated by two-layer photolithography (Fig. 1)^45^. Briefly, the channel geometries in each layer were defined by patterning the SU-8 photoresist (Microchem) through the photomask on a silicon wafer (Silicon, Inc., ID). The first layer was used to define the cell-docking structure (3 μm thick). The second layer was used to define the flow channels (70 μm thick). The SU-8 master was then molded by polydimethylsiloxane (PDMS)(Sylgard 184, Dow Corning) by soft-lithography to create the negative replica. Inlets and outlets were punched out of the PDMS replica. The PDMS replica was then bonded to a glass slide by air plasma treatment. Before chemotaxis experiment, the microfluidic device was coated with fibronectin (0.25 mg/mL, BD Biosciences) for 1 hr followed by 0.4% BSA blocking for another 1 hr all at room temperature. A new microfluidic device was used for each experiment.

### Cell preparation

Whole blood from healthy donors was collected at the Victoria General Hospital in Winnipeg under an approved ethics protocol. Informed consent form was obtained from all participants by the recruiting staff employed at the Victoria General Hospital following the procedures approved by the ethics board. Participants were given the opportunity to review the consent form in details and discuss with the recruiting staff for questions before they answer the questions in the consent form and provide their consenting signatures in the form. Neutrophils were isolated using a magnetic negative selection kit (EasySep Direct, STEMCELL Technologies, Inc.). Isolated neutrophils were suspended in RPMI medium with 0.4% BSA and kept in a 37 °C incubator before used for experiments within 8 hrs.

### FGF23 treatment and cell migration experiment

Neutrophils were divided into the test group and the control group. The test group was pre-treated with 100 ng/mL FGF23 (R&D Systems Inc.) for 1 hr in a 37 °C incubator. The cells were washed and re-suspended in migration medium (RPMI-1640 with 0.4% BSA) before used for cell migration experiments. The FGF23 pre-treated cells were tested for their migration in a 100 nM *f*MLP gradient (Sigma-Aldrich) or a 100 nM *f*MLP gradient with a 100 ng/mL FGF23 uniform background, and the control cells were tested in a 100 nM *f*MLP gradient. Each set of experiments of the three conditions was done in parallel on a single microfluidic device. The experiments were performed using both the C^3^-Chip and the D^3^-Chip. In a separate set of experiments, the control cells were tested for their migration in a 100 nM *f*MLP gradient or a 100 nM *f*MLP gradient with a 100 ng/mL FGF23 uniform background using the C^3^-Chip or the D^3^-Chip. All solutions were prepared in the migration medium. FITC-Dextran (10 kDa, final concentration of 5 μM, Sigma-Aldrich) was added to the chemoattractant solutions for gradient measurement using an inverted fluorescence microscope (Nikon Ti-U). After neutrophils were seeded on the fibronectin-coated microfluidic channels, different gradient configurations were applied to each test unit. Cell migration was recorded by time-lapse microscopy using an inverted fluorescence microscope (Nikon Ti-U) with a stage chamber to control the temperature at 37 °C. Time-lapse differential interference contrast (DIC) images were acquired at 6 frames/min for 15 min.

### Cell migration data analysis

#### Single parameter analysis

Time-lapse images were tracked and analyzed using the “Manual Tracking” plug-in in NIH ImageJ. Several migration parameters were calculated based on the tracking data including chemotactic index (CI), which is the ratio of the displacement of cells toward the gradient to the total migration distance; flowtactic index (FI), which is the ratio of the displacement of cells along the flow direction to the total migration distance; and the migration speed (V), calculated as the ratio of total migration distance to the experiment period. The net cell movement along the flow direction is defined as positive, and against the flow as negative for the FI calculation.

In this study, we further used chemotactic entropy (CE) to describe the level of disorder of cell migration direction in response to a chemoattractant gradient.

1 $$CE=-{\sum }_{i=1}^{12}\{|{\rm{\Delta }}{\theta }_{i}|\times \frac{{n}_{i}}{N}\times \,\mathrm{log}\,\frac{{n}_{i}}{N}\}$$

here *n*_*i*_ is the number of cells fall in the *i* ^*th*^ $\frac{\pi }{6}$ angle interval; *i* is an integer from 1 to 12 as the index label for the 12 angle intervals; *N* is the total number of cells. The first angle interval (*i* = 1) is set between $2\times \pi -\frac{\pi }{12}$ and $\frac{\pi }{12}$; the following angle intervals rotate counterclockwisely with increasing *i* and $\frac{\pi }{6}$ increment. Thus $\frac{{n}_{i}}{N}$ is the migration angle state density for the *i* ^*th*^ $\frac{\pi }{6}$ angle interval. $|{\rm{\Delta }}{\theta }_{i}|$ is the chemotactic weighing factor (value range between 0 and π), which is the absolute angle difference between the migration angle state (i.e. use $(i-1)\times \frac{\pi }{6}$ for the *i* ^*th*^ angle interval) and the gradient direction at $\frac{\pi }{2}$. The migration angle of each cell was defined as the angle of the connecting line between the cell’s initial and final position over the whole experiment period with respect to the positive horizontal direction. Thus, highly directional migration toward the gradient direction will show a smaller value of CE, and random migration or migration deviated from the gradient direction will show a larger value of CE.

All experiments were repeated three times. The cell migration parameters under each condition in each experiment are presented as the average value ± standard error of the mean (s.e.m.) of all cells. At least 30 cells were tracked under each condition in each experiment. The CE is presented as the average value. The data shown in the figures are from one set of representative experiments. Statistical analysis was performed using the Student’s *t*-test in Origin. *p* < 0.05 (‘*’) was considered significantly different.

#### PCA analysis

In addition, we applied the principal component analysis (PCA) for the cell tracking data. A panel of quantitative cell migration parameters was used for PCA (Table 1). The PCA was performed using the XLSTAT plug-in for Microsoft Excel (Addinsoft). Cells with or without FGF23 exposure were labeled in different colors and plotted in a 3D transformed domain of the first three principle component axes. Furthermore, the cell migration parameters were plotted as vectors to indicate their correlation in the 3D transformed domain of the first three principle component axes. The squared cosines of variables, which indicate the correlation of each cell migration parameters to the principle component axes, and the correlation matrix of variables was also calculated (Tables S1 and S2).

## Electronic supplementary material

Supplementary information
