# Supplementary material for: Fibroblast growth factor 23 weakens chemotaxis of human blood neutrophils in microfluidic devices
Source: Sci Rep. 2017 Jun 8;7:3100. doi: 10.1038/s41598-017-03210-0 (PMC5465076; doi:10.1038/s41598-017-03210-0)
Supplement: Supplementary file 1 — Supplementary information [file 41598_2017_3210_MOESM1_ESM.pdf]

Fibroblast growth factor 23 weakens chemotaxis of human blood neutrophils in microfluidic devices

Ke Yang, Hagit Peretz-Soroka, Jiandong Wu, Ling Zhu, Xueling Cui, Michael Zhang, Claudio Rigatto, Yong Liu*, Francis Lin*

Supplementary Information

Figure S1

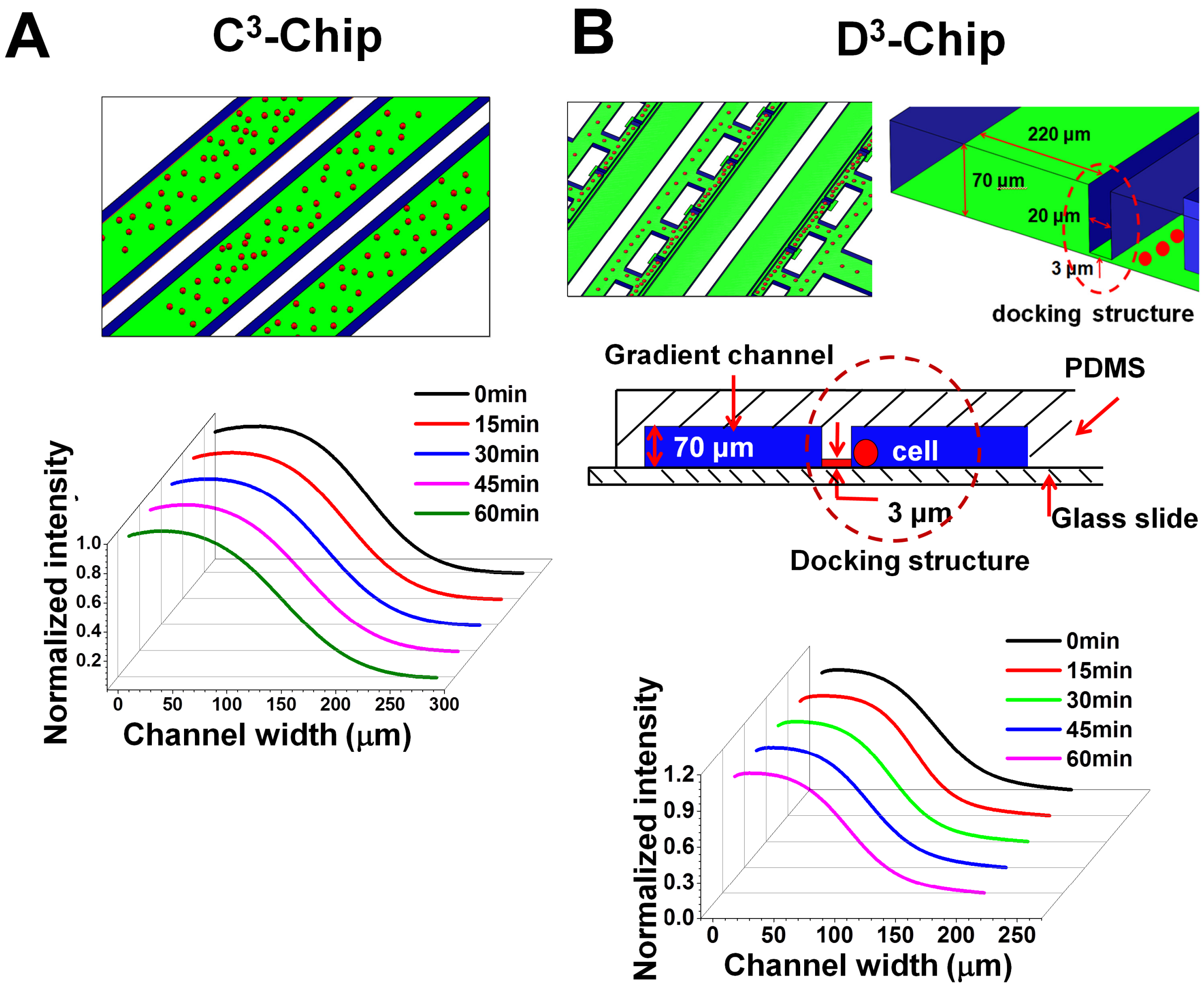

Figure S1. **Cell loading and gradient stability in the C^3-Chip and D^3-Chip.** (A) Illustration of uniform cell loading (red dots) in the 3 parallel gradient channels of the C^3-Chip. The gradient profile in each channel is stable for 60min. Gradient profiles across the channel (as measured by the normalized fluorescent intensity of FITC-Dextran additive) at different time points are plotted; (B) Illustration of cell trapping (red dots) in the 3 parallel units of the D^3-Chip. Cells are docked by the barrier channel, which is thinner than the cell diameter. Upon gradient stimulation, cells will change their morphology and crawl through the barrier channel into the gradient channel. An enlarged view of the docking structure is also shown. The side view of the docking structure is also shown. The gradient profile in each channel is stable for 60min. Gradient profiles across the channel (as measured by the normalized fluorescent intensity of FITC-Dextran additive) at different time points are plotted.

Figure S2

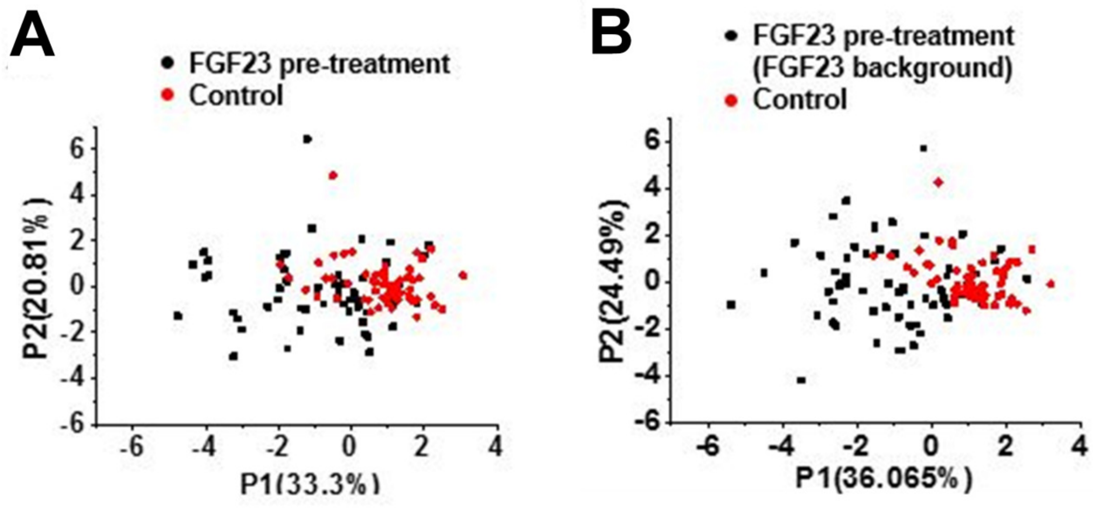

Figure S2. **P1-P2 plot of FGF23 treated cells and control cells in PCA analysis.** (A) Score plot of FGF23 pre-treated cells and un-treated control cells in a *f*MLP gradient in the 2D transformed domain of the first two principle component axes; (B) Score plot of FGF23 pre-treated cells and un-treated control cells in a *f*MLP gradient with a uniform FGF23 background in the 2D transformed domain of the first two principle component axes. The data shown are from representative experiments using the C^3-Chip.

Table S1

Squared cosines of variables

	P1	P2	P3
**FI**	0.089	0.142	**0.465**
**CI**	**0.815**	0.068	0.013
**speed**	0.006	**0.679**	0.049
**directionality**	**0.540**	0.366	0.048
**angle**	**0.386**	0.021	**0.370**
**velocity**	**0.687**	0.007	0.113
**pause number**	0.171	**0.436**	0.040
**onset time**	0.192	0.239	0.110

Correlation matrix of variables

	FI	CI	speed	directionality	angle	velocity	pause number	onset time
**FI**	**1**	-0.167	-0.163	0.078	**0.412**	-0.087	0.148	0.151
**CI**	-0.167	**1**	-0.181	**0.748**	**-0.613**	**0.656**	-0.219	-0.230
**speed**	-0.163	-0.181	**1**	**-0.402**	-0.071	0.227	**-0.454**	-0.304
**directionality**	0.078	**0.748**	**-0.402**	**1**	-0.183	**0.758**	0.053	-0.099
**angle**	**0.412**	**-0.613**	-0.071	-0.183	**1**	-0.246	0.243	0.114
**velocity**	-0.087	**0.656**	0.227	**0.758**	-0.246	**1**	-0.240	-0.271
**pause number**	0.148	-0.219	**-0.454**	0.053	0.243	-0.240	**1**	**0.437**
**onset time**	0.151	-0.230	-0.304	-0.099	0.114	-0.271	**0.437**	**1**

SI Table 1. **Squared cosines of cell migration parameters with respect to the first three principle component axes and correlation matrix of cell migration parameters.** The data shown are from representative experiments comparing chemotaxis of FGF23 pre-treated cells and the un-treated control cells in a *f*MLP gradient using the C^3-Chip.

Table S2

Squared cosines of variables

	P1	P2	P3
**FI**	0.003	0.001	**0.747**
**CI**	**0.770**	0.048	0.002
**speed**	0.001	**0.726**	0.002
**directionality**	**0.787**	0.096	0.051
**angle**	0.138	0.007	**0.651**
**velocity**	**0.778**	0.025	0.038
**pause number**	0.036	**0.526**	0.000
**onset time**	0.151	0.237	0.004

Correlation matrix of variables

	FI	CI	speed	directionality	angle	velocity	pause number	onset time
**FI**	**1**	-0.004	-0.070	0.077	**0.500**	0.011	0.002	-0.041
**CI**	-0.004	**1**	-0.155	**0.755**	**-0.410**	**0.627**	-0.090	-0.188
**speed**	-0.070	-0.155	**1**	-0.243	0.027	0.280	**-0.404**	-0.238
**directionality**	0.077	**0.755**	-0.243	**1**	-0.121	**0.824**	0.041	-0.163
**angle**	**0.500**	**-0.410**	0.027	-0.121	**1**	-0.091	0.025	0.095
**velocity**	0.011	**0.627**	0.280	**0.824**	-0.091	**1**	-0.178	-0.281
**pause number**	0.002	-0.090	**-0.404**	0.041	0.025	-0.178	**1**	0.245
**onset time**	-0.041	-0.188	-0.238	-0.163	0.095	-0.281	0.245	**1**

SI Table 2. Squared cosines of cell migration parameters with respect to the first three principle component axes and correlation matrix of cell migration parameters. The data shown are from representative experiments comparing chemotaxis of FGF23 pre-treated cells and the un-treated control cells in a *f*MLP gradient with a FGF23 uniform background using the C^3-Chip.
